# Food Reward after Bariatric Surgery and Weight Loss Outcomes: An Exploratory Study

**DOI:** 10.3390/nu14030449

**Published:** 2022-01-20

**Authors:** Erika Guyot, Julie-Anne Nazare, Pauline Oustric, Maud Robert, Emmanuel Disse, Anestis Dougkas, Sylvain Iceta

**Affiliations:** 1 Department of Endocrinology Diabetes and Nutrition, Integrated Center for Obesity, Hospices Civils de Lyon, Lyon-Sud Hospital, 69310 Pierre-Bénite, France; erikaguyot.pro@gmail.com (E.G.); emmanuel.disse@chu-lyon.fr (E.D.); 2 Centre de Recherche en Nutrition Humaine Rhône-Alpes (CRNH-RA), Laboratoire Centre Européen Nutrition et Santé (CENS), 69310 Pierre-Bénite, France; julie-anne.nazare@univ-lyon1.fr; 3 CarMeN, Unité INSERM 1060, Université Claude Bernard Lyon 1, 69310 Pierre-Bénite, France; 4 Institut Paul Bocuse Research Center, 69130 Lyon, France; anestis.dougkas@institutpaulbocuse.com; 5 School of Psychology, University of Leeds, Leeds LS2 9JT, UK; pspjo@leeds.ac.uk; 6 Department of Digestive and Bariatric Surgery, Integrated Center for Obesity, Hospices Civils de Lyon, Hôpital Edouard Herriot, 69437 Lyon, France; maud.robert@chu-lyon.fr; 7 Centre de Recherche de l’Institut Universitaire de Cardiologie et de Pneumologie de Québec, Université Laval, Québec City, QC G1V 4G5, Canada

**Keywords:** food reward, liking, wanting, food preferences, bariatric surgery, eating behavior, total weight loss

## Abstract

Changes in food preferences after bariatric surgery may alter its effectiveness as a treatment for obesity. We aimed to compare food reward for a comprehensive variety of food categories between patients who received a sleeve gastrectomy (SG) or a Roux-en-Y gastric bypass (RYGB) and to explore whether food reward differs according to weight loss. In this cross-sectional exploratory study, food reward was assessed using the Leeds Food Preference Questionnaire (LFPQ) in patients at 6, 12, or 24 months after SG or RYGB. We assessed the liking and wanting of 11 food categories. Comparisons were done regarding the type of surgery and total weight loss (TWL; based on tertile distribution). Fifty-six patients (30 SG and 26 RYGB) were included (women: 70%; age: 44.0 (11.1) y). Regarding the type of surgery, scores were not significantly different between SG and RYGB, except for ‘non-dairy products—without color’ explicit liking (*p* = 0.04). Regarding TWL outcomes, explicit liking, explicit wanting, and implicit wanting, scores were significantly higher for good responders than low responders for ‘No meat—High fat’ (post-hoc corrected *p*-value: 0.04, 0.03, and 0.04, respectively). Together, our results failed to identify major differences in liking and wanting between the types of surgery and tended to indicate that higher weight loss might be related to a higher reward for high protein-content food. Rather focus only on palatable foods, future studies should also consider a broader range of food items, including protein reward.

## 1. Introduction

Bariatric surgery is considered the most effective treatment in case of severe complicated or morbid obesity, to achieve sustained weight loss, reduce comorbidities and mortality [1,2,3]. The most commonly performed surgical techniques are sleeve gastrectomy (SG) and Roux-en-Y gastric bypass (RYGB) [4]. Both types of procedures give similar weight loss patterns at 5-year follow-up [5].

Bariatric surgery has a direct effect on food intake, including reducing ingested volume [6] and total energy intake [6,7], as well as reducing hunger and increasing satiety [8]. In addition, our recently published systematic review and meta-analyses found that food preferences change after bariatric surgery in terms of macronutrients distribution, with higher contributions of proteins and lower contributions of lipids in the total energy intake postoperatively [9]. Food preferences also change in terms of food selection with higher consumption of healthy foods and lower consumption of highly palatable foods. Finally, it changes in terms of food appreciation with an overall lower hedonic rating postoperatively compared to before the operation [9].

The attribution of a hedonic value to foods is the result of the contribution of two domains: the sensory and the reward domains. The alterations of the sensory domain (gustation and olfaction) after bariatric surgery have been examined in recent systematic reviews [10,11]. Of importance, Nielsen et al. found that patients with RYGB have a higher sweet taste sensitivity [12]. Moreover, a recent publication by our research team has demonstrated that participants with and without taste or smell alterations have different food preferences [13]. On the other hand, food reward is a constantly evolving concept that includes different models. Berridge et al. described food reward as based on two distinct components [14]. The first component is ‘liking’ and is related to the pleasure and the sensory properties of foods. The second component is ‘wanting’, which is related to the motivation to eat. In turn, wanting can be divided into two components: ‘explicit wanting’, which can be conceptualized as the conscious motivation to eat, and ‘implicit wanting’ that is more related to the subliminal level (i.e., cue-elicited motivation to eat) [15,16,17,18]. Interestingly, a post-surgery decrease in neuronal activity in brain areas corresponding to the reward system has been described [19,20,21,22,23]. Furthermore, liking and wanting scores (based on a five-point Likert type scale on the questions “How much do you want to eat this food now?” and “How much do you like this food in general?”) were diminished after RYGB for foods high in fat using food pictures displayed during an fMRI paradigm [24].

Overall, while food reward is described as dysfunctional in the case of obesity [25,26], its alterations in relation to bariatric surgery remain to be explored. Several factors including appearance, texture, taste, and the nature of foods could influence food preferences in the general population [27] and in patients after bariatric surgery [28]. However, no study explored food reward (liking and wanting) for a comprehensive range of food items postoperatively. RYGB and SG might give similar weight loss patterns at 5-year follow-up [5]. However, it has been suggested that RYGB would be more rapidly effective and lead to a higher initial weight loss and that this may be related to specific food reward changes induced by RYGB [29]. However, most of the studies so far failed to separate and explore the impact of the type of surgery on food preferences given that the analysis was conducted on only one type of surgery or by combining patients with different types of surgery. Finally, no study explored food reward in relation to weight loss status after bariatric surgery.

The aim of the present exploratory study was to compare food reward for extended various food categories between (i) patients who received SG or RYGB and (ii) according to weight loss status. We hypothesized that (i) liking and wanting scores are not different between patients with RYGB and SG and that (ii) liking and wanting scores are higher in participants with a poor weight loss response, especially regarding high carbohydrates and high fat products.

## 2. Materials and Methods

### 2.1. Subjects

From June 2018 to July 2019, participants hospitalized in our bariatric surgery tertiary care center for a routine post-bariatric surgery follow-up daycare (6, 12, or 24 months after SG or RYGB) were eligible. For this cross-sectional study, inclusion criteria were: age between 18 to 65 years old, BMI between 18.5 and 60 kg/m^2^. Patients with psychiatric comorbidities, under psychotropic treatments or having major food avoidance, as well as those unable to give their consent and understanding French were excluded. This analysis is part of a larger study (including non-operated participants) that was approved by a national ethic Committee (2017-A02953-50, on 16 January 2018) and was registered in Clinical Trials.gov (NCT03486210). Due to methodological issues (inconsistent fasting time), the control group (i.e., participants with obesity without surgery) has not been considered in the present publication.

### 2.2. Data Collection

#### 2.2.1. Food Reward

Among the various tools that exist to measure food reward, we used the Leeds Food Preference Questionnaire (LFPQ) [17], in a version adapted by Van der Meij et al. [30] as it gives a behavioral measure of food reward quantifying both explicit liking and implicit wanting. Explicit liking was measured using visual analog scales (VAS) on the question “How pleasant would it be to taste some of this food now?” Implicit wanting was computed using a forced-choice task during which a spontaneous choice must have been made between all combinations of food items presented consecutively in pairs. An implicit wanting score for a given food category is calculated from the frequency of choice and non-choice of this food category and the reaction time of the participants [18]. This score ranges between −100 and 100 and is interpreted in relation to the scores of the other food categories evaluated in the task. A positive score indicates that the considered food category was chosen more often and faster than the others. A negative score indicates the opposite.

Although the LFPQ as adapted by Van der Meij [30] was initially intended for use among the elderly, this version seemed relevant to explore food preferences among patients after bariatric surgery. In particular, it was composed of six stages which allowed us to assess liking and wanting for 11 food categories varying in nutritional composition (high/low in carbohydrates, high/low in fat, high/low in protein, dairy/nondairy, with/without fiber, with/without meat) and/or in taste (savory/sweet), appearance (with/without color, with/without variation, with/without sauce), and texture (solid/fluid) (Table 1). These food categories were combined within a task (i.e., high fat—sweet, high fat—savory, low fat—sweet, low fat—savory).

We adapted this version for its use in a French population by translating the instructions from English to French and adapting the food items to French eating habits [18]. The new set of food pictures was validated by asking 20 health professionals specialized in nutrition or dietetics and 20 non-specialized counterparts whether the food pictures correspond to what they can eat in France using a nine-point Likert-type scale where 1 represented ‘Not at all’ and 9 ‘Extremely’. Food items with a score ≤5 were considered not representative of French eating habits and were replaced. The list of food items used is available in Table 1.

#### 2.2.2. Other Covariates

Information on gender, age, and body mass index (BMI) kg/m^2^ before surgery and at assessment time point were collected based on the medical record of the patients. Patients self-reported ‘having a budget constraint to purchase food’ and ‘smoking status’. Ratings of hunger, fullness, and desire to eat were assessed immediately prior to processing the LFPQ using visual analog scales (100 mm) from ‘Not at all’ to ‘Extremely’ of the following questions: “How hungry do you feel now?”, “How full do you feel now?”, and “How strong is your desire to eat now?”. Time since the last meal was also recorded. The weight loss results were expressed as a percentage of the total weight loss (%TWL = [(Initial Weight) − (Postoperative Weight)]/[(Initial Weight)] × 100). The %TWL metric has been considered a better metric than the percentage excess weight loss because its calculation does not need to define an ideal body weight and did not differ between lower and higher baseline BMI [31] and is a better predictor of metabolic outcome [32,33].

### 2.3. Statistical Analysis

General characteristics, appetite sensations, and time since the last meal of patients were compared using independent *t*-test, Pearson’s χ^2^ test or Fisher’s exact test for normal continuous, categorical variables without, or variables with at least one expected frequency in a fourfold table less than 5, respectively. Mann–Whitney U test was performed to compare non-normal continuous variables (i.e., time since the surgery).

In the present study, patients having a wanting score <−100 and >100 in a food category were considered as outliers, and results for the food category were excluded from the analyses. Explicit liking, explicit wanting and implicit wanting scores for all the studied food categories were compared between the groups of patients (SG and RYGB) using Student’s *t*-test. We also compared liking and wanting scores between patients at 6, 12, and 24 month follow-up using one-way ANOVA. As mean and range for %TWL was expected to differ at 6, 12, and 24 months after bariatric surgery, we calculated the tertile separately for each post-surgery follow-up duration group (6, 12, and 24 months). Participants in the first tertile (<33%) were considered as low responders, in the middle tertile (33–66%) as middle responders and, in the upper tertile (>66%) as good responders. All participants were analyzed together using one-way ANOVA. Post-hoc analyses were Bonferroni corrected using the SPSS software post-hoc option and post-hoc corrected *p*-value provided for all ANOVA *p*-value < 0.1.

Data are presented as mean (SD). All the statistical analyses were conducted using SPPS software version 26.0.0.1 for Mac. All tests were two-sided and *p* < 0.05 was considered significant.

## 3. Results

### 3.1. Characteristics of the Subjects

In the study, 61 patients were initially recruited. Five participants were excluded from the study (two for antidepressant medication, one for actual major depressive disorder, and two for a follow up duration >24 months). A total of 56 participants (30 SG and 26 RYGB) were included in the present analysis. Table 2 presents the characteristics of the patients according to their surgery group.

Overall, 75% of the patients were women. Patients with RYGB were significantly older (50.5 y, SD = 9.0) compared to those with SG (38.3 y, SD = 9.6). The mean BMI before surgery of the SG group (45.5 kg/m^2^, SD = 5.9) was significantly higher compared to the RYGB group (41.4 kg/m^2^, SD = 4.4). The number of participants for each follow-up duration was similar between SG (n = 10 for 6, 12, and 24 months) and RYGB (n = 10 for 6 months, n = 7 for 12 months, and n = 9 for 24 months; *p* = 0.862). The other parameters, including %TWL, appetite sensations scores, were not significantly different between the two groups of surgery.

### 3.2. Relationship between Liking and Wanting for Foods and Type of Bariatric Surgery

**Figure 1:**
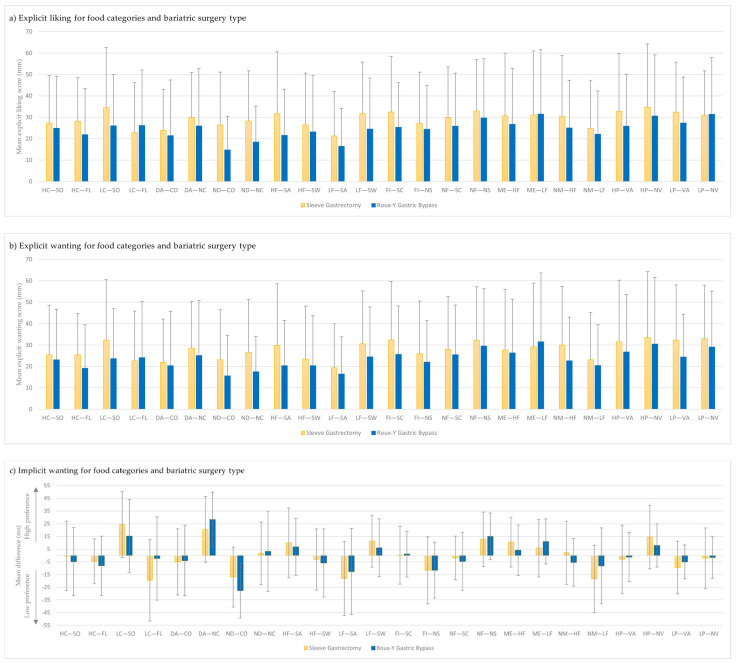
Relationship between (**a**) explicit liking, (**b**) explicit wanting and (**c**) implicit wanting for foods and bariatric surgery type. Food categories tested with the Leeds Food Preference questionnaire are indicated on the *x*-axis. Abbreviations: CO, color; DA, dairy; FI, fiber; FL, fluid; HC, high carbohydrate; HF, high fat; HP, high protein; LC, low carbohydrate; LF, low fat; LP, low protein; ME, meat; NC, no color; ND, non-dairy; NF, no fiber; NM, no meat; NS, no sauce; NV, no variation; OB: non-operative patients with obesity; SA, savory; SC, sauce; SO, solid; SW, sweet; VA, variation. Data are presented as mean (SD).

Explicit liking, explicit and implicit wanting scores regarding surgery type are illustrated in Figure 1 and detailed in Supplementary Material Table S1. Considering all the studied food categories, scores were not significantly different between SG and RYGB, except for ‘non-dairy products—without color’ explicit liking (26.5, SD = 24.7 for SG and 14.8, SD = 15.7 for RYGB; *p* = 0.038). We also observed a trend for a significant difference for ‘non-dairy products—without color’ implicit wanting (−17.0, SD = 23.7 for SG and −27.6, SD = 21.7 for RYGB; *p* = 0.089), ‘non-dairy products—no color’ explicit liking (28.3, SD = 23.5 for SG and 18.5, SD = 16.7 for RYGB; *p* = 0.087), and for foods ‘low in carbohydrates—fluid’ implicit wanting (−19.5, SD = 32.0 for SG and −2.3, SD = 32.8 for RYGB; *p* = 0.053). A negative implicit wanting score means that the food category items were more often rejected relatively to the items in the other food categories. Moreover, explicit liking, explicit wanting and implicit wanting scores for each follow-up duration (6, 12, and 24 months) were not significantly different for any of the food categories (data not shown).

### 3.3. Relationship between Liking and Wanting for Foods Regarding Weight Loss Outcomes

Regarding comparison between %TWL groups, Bonferroni post-hoc test revealed that differences were significant only between Low and Good responders. Detailed results are available in Table 3, Table 4 and Table 5 (characteristics of the subjects for each tertile are presented in Supplementary Material Table S2).

Scores were significantly higher for good responders than Low responders for ‘No meat—High fat’ and this for the three types of measures, explicit liking, explicit wanting and implicit wanting (post-hoc corrected *p*-value: 0.037, 0.033 and 0.043, respectively). Regarding ‘High protein–Variation’ items, explicit liking was higher in good responders than in Low responders (post-hoc corrected *p*-value = 0.030).

Regarding implicit wanting scores, more differences were found between good and low responders. In addition to the higher scores mentioned above for the ‘No meat—High fat’ category, good responders also had significant higher scores than Low responders for ‘Low carb—Solid’ (post-hoc corrected *p*-value = 0.011) and tended to be significantly higher for ‘High protein—No variation’ items (post-hoc corrected *p*-value = 0.061). A trend to significant difference was also observed for ‘Low protein—Variation’ items with good responders having lower scores than low responders. Taking together, these results suggest that participants with higher %TWL tended to present higher implicit wanting for food items with higher protein content.

## 4. Discussion

The present exploratory study aimed to investigate thoroughly liking and wanting for food items (i.e., food reward components) in participants after bariatric surgery using a computerized behavioral task. The originality of this study relies on the use of a wide range of food items varying in nutrition composition, appearance, taste, and texture and on its comparative design considering the types of surgery (SG and RYGB) and the weight loss outcome.

The present work is among the few studies using a distinct comparative design towards the two most common types of bariatric surgery. Regarding socio-demographic data, participants with SG or RYGB did not differ significantly except for age (SG = 38.3 years and RYGB = 50.5 years, *p* < 0.001). Age and gender influence on taste has been documented and might have influenced our results [34]. Although statistically significant, this difference appears to be of small clinical relevance given that all patients belong to the same middle-age category and may not influence food preferences. Our findings suggest that there is no major difference between the two types of surgery regarding liking and wanting for all foods categories assessed. This is in accordance with studies, showing a similar decrease in preference for fat [35] and liking for high-calorie dense foods [36], as well as an increase in diet quality [37] and dietary intake [38] after both SG and RYGB. However, another study showed a decrease in the hedonic rating for high fat after SG and RYGB, but the liking of high sugar remained unchanged after RYGB [35]. On the other hand, a recent study published by Lewis et al. suggests that RYGB tends to be associated with lower enjoyment and craving for highly palatable foods than SG [39]. RYGB induces more dumping syndrome than SG [40], and thus the digestive discomfort caused by the consumption of foods rich in carbohydrates may have resulted in stronger conditioned taste avoidance in RYGB than in SG. Although this is a frequent issue in clinical practice, our results do not indicate any relevant changes in relation to carbohydrates liking or wanting, in any form (i.e., solid or liquid).

Considering weight loss outcomes (assessed by %TWL), comparisons of explicit liking and explicit and implicit wanting scores between low and good weight loss responders suggest differences in reward depending on food protein content. In our study, participants with a better weight loss trajectory tend to prefer foods with higher protein content. Interestingly, these results could be explained within the framework of the protein leverage hypothesis. This hypothesis was primarily developed to explain overeating in the context of obesity [41,42,43]. This theory assumes that protein intake is prioritized over carbohydrates and fat intake. Accordingly, participants eat until their protein needs are met, regardless of the energy content of the food. Thus, participants with higher weight loss may have a stronger desire (and consumption) for protein and therefore, consume less palatable products (with high energy density but low protein content). Further confirmation of such findings in a prospective study would be of importance, especially to determine whether a higher pre-surgery protein wanting causes a greater weight loss or whether such modifications appear after bariatric surgery.

In contrast to our expectations, patients with lower weight loss did not show higher liking or wanting for products rich in fat or carbohydrates, except for ‘Low carb—Solid’ implicit wanting. A systematic review showed that food reward decreased during dietary, exercise, pharmacological, cognitive, and behavioral/multidisciplinary weight-loss interventions [44]. This suggests that more than the bariatric surgery itself, the weight loss and therefore the energy imbalance might have an impact on the motivation to eat and food choices. Most patients experience a ‘honeymoon’ in the first post-operative year (12–18 months), where they have a reduced appetite, limiting portion sizes, and disinterest in foods [45], which is concomitant with the great and rapid weight loss phase. Our results might be explained considering the time points of assessment reflecting the time since surgery. Indeed, in a previous study, we observed that participants had a higher appreciation of highly palatable foods after 2 years of follow-up than those having been operated on more recently [13]. Other studies also suggest a return of dietary preferences to prior patterns with increasing time since surgery [37,46,47]. Using the Leeds Food Preference Questionnaire, Oustric et al. also observed such a backward step after weight loss programs [48]. Prospective and long-term assessments (after 2 years of follow-up) of food rewards are necessary to better understand if those modifications are sustained and/or if they are related to the weight regain that occurs in 20% of patients within 1 to 3 years after surgery [49].

Some limitations must be considered, especially the cross-sectional design of the study and the small sample size. The transversal design does not allow us to test the causal effect of bariatric surgery on the differences of food reward found between lower weight loss responders and good or normal weight loss responders. The relatively small sample size and the sampling method (routine daycare) is also an important limitation, especially considering the important number of statistical analyses conducted. Larger sample sizes are needed to achieve the required power to apply a proper correction for multiple testing. Another limitation is the lack of controlled laboratory conditions when measuring food preferences as the visits occurred in the hospitalization unit and the time of arrival of the patients were based on their pre-registered clinical consultancy, which prevented rigorously standardizing fasting status, appetite levels, and time since the last meal in patients. However, when comparing the type of surgery or the weight loss trajectory status, the time from last meal or appetite sensations did not differ between groups. Our results showed a significant difference in age between patients with SG and RYGB (38 vs. 51 years old respectively). Finally, food preferences could have been influenced by other parameters, such as the socio-cultural background and ethnicity, which could be considered in future research given their influence on food choices [50,51]. Despite these limitations, our study, by addressing a wider range of food items, highlights the need to consider adding protein items in future research on food rewards, especially in the bariatric surgery field.

## 5. Conclusions

To conclude, liking and wanting for a comprehensive range of food items were not different in participants with either SG or RYGB. Wanting for protein items might be related to the weight loss response after bariatric surgery, while no such association was found for usual palatable foods. Adding protein items to usual palatable food items could be clinically relevant to identify postoperative alterations in food reward and guide caregivers to give personalized advice to patients in the context of precision bariatric medicine.

## Figures and Tables

**Table 1:** Dichotomous food categories tested with the Leeds Food Preference questionnaire.

Task	Studied Dimensions	Combined Food Categories
1	High carbohydrates (HC)/Low carbohydrates (LC) Solid (SO)/Fluid (FL)	HC—SO HC—FL LC—SO LC—FL
2	Dairy (DA)/Non-dairy (ND) Color (CO)/No color (NC)	DA—CO DA—NC ND—CO ND—NC
3	High fat (HF)/Low fat (LF) Savory (SA)/Sweet (SW)	HF—SA HF—SW LF—SA LF—SW
4	Fiber (FI)/No fiber (NF) Sauce (SC)/No sauce (NS)	FI—SC FI—NS NF—SC NF—NS
5	Meat (ME)/No meat (NM) High fat (HF)/Low fat (LF)	ME—HF ME—LF NM—HF NM—LF
6	High protein (HP)/Low protein (LP) Variation (VA)/No variation (NV)	HP—VA HP—NV LP—VA LP—NV

The tasks were administered to patients in a random order.

**Table 2:** Socioeconomic and behavioral characteristics of the 56 included patients according to their operative status.

	All n = 56	SG n = 30	RYGB n = 26	*p*-Value
Sociodemographic data				
Women (%)	75.0 (n = 42)	76.7 (n = 23)	73.1(n = 19)	0.757
Age (year)	44.0 (11.1)	38.3 (9.6)	50.5 (9.0)	<0.001
Smoking status (%)	19.6 (n = 11)	20.0 (n = 6)	19.2 (n = 5)	0.942
Food budget constraint (%)	14.3	10.0	19.2	0.451 ^a^
Anthropometric data				
BMI Before surgery(kg/m^2^)	43.6 (6.0)	45.5 (5.9)	41.4 (4.4)	0.011
Body weight (kg)	87.8 (17.8)	88.2 (20.3)	87.3 (14.7)	0.843
BMI (kg/m^2^) ^†^	31.4 (4.5)	31.5 (4.5)	31.2 (4.6)	0.846
%TWL	27.7 (7.3)	30.6 (6.3)	24.3 (7.0)	<0.001
Appetite sensations				
Mean hunger (mm)	21.0 (25.7)	25.2 (28.7)	16.2 (21.5)	0.196
Mean fullness (mm)	68.3 (28.0)	68.7 (27.6)	67.8 (29.0)	0.909
Mean desire to eat (mm)	22.2 (24.8)	25.6 (28.2)	18.2 (19.8)	0.252
Time since last meal (min)	134 (214)	154 (237)	109 (184)	0.434

Abbreviations: SG: group with a sleeve gastrectomy; RYGB: group of patients with a Roux-en-Y gastric bypass; BMI: Body Mass Index; %TWL: Percentage of total weight loss. ^†^ BMI at the follow-up visit considered for this study. Data are presented as mean (SD), or percentage (number). *p* values are for the differences between patients with SG and RYGB on the basis of Student’s *t*-test and Pearson’s χ^2^ test; ^a^ indicates that Fisher’s exact test or Mann–Whitney U test were used.

**Table 3:** Relationship between explicit liking for foods regarding percentage of total weight loss tertile groups.

	Low Responders <33%			Middle Responders 33–66%			Good Responders >66%			ANOVA *p*-Value
	n = 18			n = 19			n = 19			
	Mean		SD	Mean		SD	Mean		SD	
High carb—Solid	22.2	±	20.0	30.1	±	26.9	26.1	±	21.9	0.581
High carb—Fluid	19.2	±	15.7	29.1	±	24.5	27.2	±	21.2	0.323
Low carb—Solid	21.4	±	23.6	30.4	±	25.5	39.5	±	28.1	0.113
Low carb—Fluid	20.3	±	18.2	30.8	±	31.4	21.9	±	21.3	0.373
Dairy—Color	17.0	±	14.5	22.3	±	23.9	28.5	±	25.7	0.303
Dairy—No color	21.1	±	14.8	28.6	±	27.2	33.7	±	26.0	0.290
Non-dairy—Color	16.6	±	19.1	20.4	±	19.2	26.0	±	26.0	0.433
Non-dairy—No color	19.3	±	19.0	20.2	±	18.8	31.4	±	23.8	0.147
High fat—Savory	19.0	±	23.5	25.2	±	22.0	36.2	±	30.2	0.126
High fat—Sweet	19.5	±	20.1	26.2	±	24.5	28.8	±	29.8	0.521
Low fat—Savory	9.7	±	10.1	22.4	±	20.6	24.6	±	22.3	0.040 ^a^
Low fat—Sweet	22.8	±	19.7	29.3	±	27.4	32.7	±	24.4	0.458
Fiber—Sauce	21.3	±	18.1	30.4	±	26.4	35.3	±	25.0	0.196
Fiber—No sauce	19.2	±	13.6	29.1	±	26.2	29.0	±	24.4	0.309
No fiber—Sauce	21.5	±	19.5	25.9	±	24.1	36.1	±	26.9	0.166
No fiber—No sauce	27.6	±	26.3	30.5	±	27.3	36.0	±	23.6	0.602
Meat—High fat	18.7	±	22.6	28.6	±	27.2	38.7	±	30.2	0.087 ^b^
Meat—Low fat	22.5	±	24.1	31.5	±	30.6	39.3	±	32.9	0.232
No meat—High fat	18.4	±	18.8	25.7	±	21.3	39.3	±	31.5	0.038 ^c^
No meat—Low fat	16.9	±	15.5	25.6	±	23.1	27.9	±	23.2	0.259
High protein—Variation	18.6	±	22.9	29.2	±	25.8	40.3	±	25.1	0.035 ^d^
High protein—No variation	23.9	±	29.1	31.2	±	27.2	42.9	±	28.7	0.129
Low protein—Variation	24.6	±	20.2	30.0	±	22.7	35.2	±	24.0	0.366
Low protein—No variation	24.3	±	21.3	32.3	±	25.0	36.6	±	23.1	0.274

^a^: post-hoc analyses indicate a trend for a significant difference only between low and good responders with a Bonferroni corrected *p*-value = 0.055; ^b^: post-hoc analyses indicate a trend for a significant difference only between low and good responders with a Bonferroni corrected *p*-value = 0.084; ^c^: post-hoc analyses indicate a significant difference only between low and good responders with a Bonferroni corrected *p*-value = 0.037; ^d^: post-hoc analyses indicate a significant difference only between low and good responders with a Bonferroni corrected *p*-value = 0.030.

**Table 4:** Relationship between explicit wanting for foods regarding percentage of total weight loss tertile groups.

	Low Responders <33%			Middle Responders 33–66%			Good Responders >66%			ANOVA *p*-Value
	n = 18			n = 19			n = 19			
	Mean		SD	Mean		SD	Mean		SD	
High carb—Solid	20.6	±	21.5	29.1	±	26.5	23.2	±	21.4	0.532
High carb—Fluid	17.4	±	15.7	26.5	±	23.9	23.4	±	19.1	0.376
Low carb—Solid	19.9	±	22.8	26.6	±	24.4	38.0	±	29.1	0.103
Low carb—Fluid	18.5	±	18.6	28.2	±	31.4	22.9	±	21.5	0.483
Dairy—Color	14.8	±	12.8	21.5	±	24.4	26.7	±	26.5	0.288
Dairy—No color	21.1	±	16.7	29.4	±	28.8	29.8	±	23.0	0.464
Non-dairy—Color	14.4	±	16.8	21.4	±	20.4	22.9	±	26.2	0.460
Non-dairy—No color	17.4	±	16.6	20.2	±	21.3	29.4	±	25.0	0.215
High fat—Savory	17.0	±	21.8	24.3	±	23.0	34.6	±	29.7	0.110
High fat—Sweet	18.1	±	21.1	23.8	±	24.1	23.9	±	26.6	0.706
Low fat—Savory	9.2	±	8.5	21.2	±	21.0	23.2	±	22.1	0.052 ^a^
Low fat—Sweet	23.2	±	21.1	27.9	±	25.9	32.0	±	25.1	0.547
Fiber—Sauce	22.8	±	20.0	28.6	±	27.5	36.1	±	26.9	0.279
Fiber—No sauce	16.9	±	13.1	27.8	±	26.9	27.3	±	23.5	0.249
No fiber—Sauce	19.5	±	19.0	26.6	±	25.3	34.2	±	25.0	0.169
No fiber—No sauce	25.8	±	23.2	31.2	±	28.5	35.7	±	24.9	0.510
Meat—High fat	18.4	±	22.3	28.0	±	27.3	34.3	±	28.4	0.189
Meat—Low fat	22.4	±	26.7	30.7	±	32.1	37.2	±	32.4	0.343
No meat—High fat	16.6	±	17.1	25.7	±	22.1	36.9	±	29.2	0.038 ^b^
No meat—Low fat	14.3	±	13.6	25.2	±	23.2	25.8	±	22.3	0.168
High protein—Variation	18.1	±	22.3	32.0	±	31.6	37.4	±	26.0	0.092 ^c^
High protein—No variation	21.8	±	27.6	33.1	±	32.0	40.8	±	30.6	0.166
Low protein—Variation	22.9	±	18.5	30.1	±	25.8	32.8	±	25.0	0.423
Low protein—No variation	24.0	±	19.7	32.9	±	29.8	36.3	±	24.8	0.321

^a^: post-hoc analyses indicate a trend for a significant difference only between low and good responders with a Bonferroni corrected *p*-value = 0.073; ^b^: post-hoc analyses indicate a significant difference only between low and good responders with a Bonferroni corrected *p*-value = 0.033; ^c^: all *p*-value for the post-hoc analyses were >0.1 after Bonferroni correction.

**Table 5:** Relationship between implicit wanting for foods regarding percentage of total weight loss tercile groups.

	Low Responders <33%			Middle Responders 33–66%			Good Responders >66%			ANOVA *p*-Value
	n = 18			n = 19			n = 19			
	Mean		SD	Mean		SD	Mean		SD	
High carb—Solid	0.94	±	27.62	−2.16	±	30.98	−5.98	±	22.97	0.744
High carb—Fluid	−7.82	±	21.69	−3.84	±	21.92	−6.92	±	18.35	0.828
Low carb—Solid	15.84	±	27.92	9.60	±	30.85	34.78	±	15.74	0.011 ^a^
Low carb—Fluid	−8.96	±	33.35	−3.60	±	34.57	−21.88	±	30.60	0.222
Dairy—Color	−2.95	±	17.49	−7.88	±	33.79	−2.74	±	26.68	0.804
Dairy—No color	23.14	±	29.23	26.66	±	24.16	22.36	±	18.97	0.846
Non-dairy—Color	−24.27	±	29.86	−13.89	±	21.12	−27.71	±	15.79	0.163
Non-dairy—No color	4.08	±	24.58	−4.89	±	29.03	8.10	±	29.33	0.345
High fat—Savory	2.86	±	28.82	2.55	±	16.86	19.26	±	25.88	0.071 ^b^
High fat—Sweet	−4.41	±	30.22	0.13	±	24.89	−8.23	±	20.57	0.619
Low fat—Savory	−11.87	±	38.09	−18.35	±	28.48	−16.33	±	28.15	0.819
Low fat—Sweet	15.41	±	23.65	6.61	±	20.30	5.30	±	20.49	0.321
Fiber—Sauce	0.79	±	20.09	3.39	±	17.73	−2.34	±	24.25	0.705
Fiber—No sauce	−14.02	±	26.57	−4.94	±	22.10	−16.39	±	23.48	0.317
No fiber—Sauce	−6.13	±	22.25	−7.36	±	20.55	3.86	±	15.33	0.164
No fiber—No sauce	18.33	±	22.80	8.91	±	16.04	14.88	±	20.46	0.359
Meat—High fat	11.10	±	22.44	1.79	±	20.95	10.32	±	15.20	0.287
Meat—Low fat	11.94	±	18.73	4.98	±	24.06	7.98	±	18.81	0.605
No meat—High fat	−11.69	±	20.54	0.27	±	27.73	6.46	±	13.73	0.046 ^c^
No meat—Low fat	−5.49	±	32.93	−10.29	±	29.78	−24.75	±	18.28	0.095 ^d^
High protein—Variation	−5.30	±	18.12	−3.27	±	31.69	1.89	±	18.25	0.635
High protein—No variation	5.25	±	18.82	7.18	±	22.66	21.69	±	20.87	0.038 ^e^
Low protein—Variation	−1.93	±	17.00	−5.17	±	19.67	−14.85	±	14.16	0.064 ^f^
Low protein—No variation	1.98	±	16.08	1.26	±	25.70	−8.73	±	17.68	0.206

^a^: post-hoc analyses indicate a significant difference only between low and good responders with a Bonferroni corrected *p*-value = 0.011; ^b^: all *p*-value for the post-hoc analyses were > 0.1 after Bonferroni correction; ^c^: post-hoc analyses indicate a significant difference only between low and good responders with a Bonferroni corrected *p*-value = 0.043; ^d^: all *p*-value for the post-hoc analyses were > 0.1 after Bonferroni correction; ^e^: post-hoc analyses indicate a trend for a significant difference only between low and good responders with a Bonferroni corrected *p*-value = 0.061; ^f^: post-hoc analyses indicate a trend for a significant difference only between low and good responders with a Bonferroni corrected *p*-value = 0.076.

## Data Availability

The data presented in this study are available on reasonable request from the corresponding author. The data are not publicly available.

## Supplementary Materials

The following are available online at https://www.mdpi.com/article/10.3390/nu14030449/s1, Table S1: Relationship between liking and wanting for foods and bariatric surgery type; Table S2: Socioeconomic and behavioral characteristics by total weight loss tertile.
