# Supplementary material for: Food Reward after Bariatric Surgery and Weight Loss Outcomes: An Exploratory Study
Source: Nutrients. 2022 Jan 20;14(3):449. doi: 10.3390/nu14030449 (PMC8840022; doi:10.3390/nu14030449)
Supplement: Supplementary file 1 [file nutrients-14-00449-s001.zip › nutrients-1563990-supplementary.pdf]

Alterations in food reward regarding bariatric surgery type and weight loss outcomes: an exploratory study.

Erika Guyot[1,2,3], Julie-Anne Nazare[2], Pauline Oustric[4], Maud Robert[5], Emmanuel Disse[1,2], Anestis Dougkas[3,†] and Sylvain ICETA[1,6,†,*]

1. Department of Endocrinology Diabetes and Nutrition - Integrated Center for Obesity Hospices Civils de Lyon, Lyon-Sud Hospital, 69310, Pierre-Bénite, France; +33 (0)4 78 86 57 86. erikaguyot.pro@gmail.com, emmanuel.disse@chu-lyon.fr
2. Centre de Recherche en Nutrition Humaine Rhône-Alpes (CRNH-RA), Unité INSERM 1060, Laboratoire Centre Européen Nutrition et Santé (CENS), CarMeN, Université Claude Bernard Lyon 1, 165, Chemin du Grand Revoyet, 69310, Pierre-Bénite, France; +33 (0)4 26 23 59 17. julie-anne.nazare@univ-lyon1.fr
3. Institut Paul Bocuse Research Center, 69130, Ecully, France; +33 (0)4 72 18 02 20. anestis.dougkas@institutpaulbocuse.com
4. School of Psychology, University of Leeds, Leeds, LS2 9JT, UK; +44 (0113) 343 3738. pspjo@leeds.ac.uk
5. Department of Digestive and Bariatric Surgery, Integrated Center for Obesity, Hospices Civils de Lyon, Hôpital Edouard Herriot, 69437 Lyon, France; CarMeN Laboratory, INSERM 1060, Lyon, France; Université Claude Bernard Lyon 1, Lyon, France; +33 (0)4 72 11 62 63. maud.robert@chu-lyon.fr
6. Centre de Recherche de l'Institut Universitaire de Cardiologie et de Pneumologie de Québec-Université Laval, 2725, Chemin Sainte-Foy, Quebec, QC G1V 4G5, Canada; +1 (418) 656-8711. sylvain.iceta.1@ulaval.ca

* These authors contributed equally to this work and shared last co-authorship

* Correspondence: sylvain.iceta.1@ulaval.ca; Tel.: +1 (418) 656-8711

Table S1 : Relationship between liking and wanting for foods and bariatric surgery type.
Table S2 : Socioeconomic and behavioral characteristics by Total Weight Loss tercile.

Table S1 : Relationship between liking and wanting for foods and bariatric surgery type

	SG n = 30	RYGB n = 26	Student-t test p value
**Explicit liking**			
High carb – Solid	27.4 ± 22.1	24.9 ± 24.3	0.69
High carb – Fluid	28.2 ± 20.4	21.9 ± 21.4	0.27
Low carb – Solid	34.5 ± 28.2	26.0 ± 24.0	0.24
Low carb – Fluid	22.9 ± 23.4	26.2 ± 25.9	0.61
Dairy – Color	23.9 ± 19.1	21.8 ± 25.9	0.69
Dairy – No color	29.8 ± 21.1	26.0 ± 26.9	0.56
Nondairy – Color	26.5 ± 24.7	14.8 ± 15.7	**0.04**
Nondairy – No color	28.3 ± 23.5	18.5 ± 16.7	0.09
High fat – Savory	31.6 ± 29.0	21.6 ± 21.5	0.15
High fat – Sweet	26.4 ± 24.2	23.2 ± 26.3	0.64
Low fat – Savory	21.3 ± 20.7	16.4 ± 17.8	0.36
Low fat – Sweet	31.7 ± 24.1	24.5 ± 23.8	0.27
Fiber – Sauce	32.5 ± 26.0	25.3 ± 20.9	0.27
Fiber – No sauce	27.1 ± 24.0	24.5 ± 20.5	0.66
No fiber – Sauce	29.8 ± 23.8	25.9 ± 24.8	0.55
No fiber – No sauce	32.9 ± 24.0	29.7 ± 27.6	0.64
Meat – High fat	30.7 ± 29.3	26.8 ± 26.0	0.60
Meat – Low fat	31.1 ± 29.9	31.5 ± 30.2	0.96
No meat – High fat	30.4 ± 28.5	25.1 ± 22.2	0.44
No meat – Low fat	24.8 ± 22.2	22.2 ± 20.1	0.64
High protein – Variation	32.8 ± 27.1	25.9 ± 24.3	0.32
High protein – No variation	34.7 ± 29.5	30.6 ± 28.6	0.60
Low protein – Variation	32.4 ± 23.3	27.3 ± 21.5	0.41
Low protein – No variation	31.0 ± 20.7	31.4 ± 26.5	0.95
**Explicit wanting**			
High carb – Solid	25.4 ± 23.2	23.2 ± 23.5	0.72
High carb – Fluid	25.5 ± 19.3	19.2 ± 20.5	0.24
Low carb – Solid	32.3 ± 28.3	23.6 ± 23.4	0.22
Low carb – Fluid	22.6 ± 23.3	24.1 ± 26.2	0.82
Dairy – Color	21.9 ± 20.2	20.4 ± 25.4	0.81
Dairy – No color	28.6 ± 21.8	25.1 ± 25.7	0.59
Nondairy – Color	23.2 ± 23.4	15.7 ± 18.8	0.20
Nondairy – No color	26.7 ± 24.6	17.5 ± 16.6	0.12
High fat – Savory	29.8 ± 28.8	20.4 ± 21.1	0.17
High fat – Sweet	23.5 ± 24.6	20.4 ± 23.3	0.64
Low fat – Savory	19.4 ± 20.6	16.5 ± 17.4	0.58
Low fat – Sweet	30.6 ± 24.7	24.5 ± 23.2	0.35
Fiber – Sauce	32.4 ± 27.3	25.6 ± 22.6	0.32
Fiber – No sauce	25.9 ± 24.6	22.0 ± 19.4	0.53
No fiber – Sauce	28.1 ± 24.5	25.5 ± 23.1	0.69

No fiber – No sauce	32.3 ± 24.9	29.6 ± 26.6	0.70
Meat – High fat	27.6 ± 28.2	26.4 ± 25.0	0.86
Meat – Low fat	29.1 ± 29.8	31.6 ± 32.1	0.77
No meat – High fat	30.0 ± 27.3	22.6 ± 20.5	0.27
No meat – Low fat	23.2 ± 22.0	20.4 ± 19.1	0.62
High protein – Variation	31.6 ± 28.7	26.8 ± 26.8	0.52
High protein – No variation	33.5 ± 30.7	30.5 ± 31.1	0.21
Low protein – Variation	32.3 ± 25.8	24.5 ± 19.9	0.72
Low protein – No variation	33.0 ± 24.9	29.1 ± 26.0	0.57
**Implicit wanting**			
High carb – Solid	-0.4 ± 27.5	-4.9 ± 26.8	0.54
High carb – Fluid	-4.5 ± 17.7	-8.1 ± 23.4	0.51
Low carb – Solid	24.4 ± 25.9	15.3 ± 28.8	0.22
Low carb – Fluid	-19.5 ± 32.0	-2.3 ± 32.8	0.05
Dairy – Color	-5.1 ± 26.0	-4.0 ± 27.7	0.88
Dairy – No color	20.4 ± 25.8	28.3 ± 21.5	0.23
Nondairy – Color	-17.0 ± 23.7	-27.6 ± 21.7	0.09
Nondairy – No color	1.6 ± 24.7	3.3 ± 31.6	0.83
High fat – Savory	10.0 ± 27.6	6.8 ± 22.4	0.66
High fat – Sweet	-3.1 ± 24.1	-5.9 ± 26.9	0.69
Low fat – Savory	-18.2 ± 29.3	-12.6 ± 33.8	0.51
Low fat – Sweet	11.2 ± 20.5	6.0 ± 22.8	0.38
Fiber – Sauce	0.3 ± 22.8	1.1 ± 18.1	0.88
Fiber – No sauce	-11.6 ± 26.4	-11.6 ± 22.0	0.10
No fiber – Sauce	-1.9 ± 17.2	-4.6 ± 22.8	0.62
No fiber – No sauce	12.7 ± 21.4	15.1 ± 18.4	0.67
Meat – High fat	10.5 ± 19.6	4.1 ± 19.8	0.23
Meat – Low fat	5.8 ± 22.7	11.0 ± 17.7	0.35
No meat – High fat	2.1 ± 24.8	-2.9 ± 19.4	0.41
No meat – Low fat	-18.4 ± 26.5	-8.1 ± 29.9	0.18
High protein – Variation	-2.9 ± 26.8	-1.3 ± 19.3	0.79
High protein – No variation	14.6 ± 25.1	7.9 ± 17.1	0.26
Low protein – Variation	-9.5 ± 20.7	-5.0 ± 13.4	0.34
Low protein – No variation	-2.2 ± 23.8	-1.6 ± 16.5	0.92

SG : Sleeve gastrectomy; RYGB : Roux en Y gastric bypass; results are expressed in mean ± SD

Table S2 : Socioeconomic and behavioral characteristics by Total Weight Loss tercile

	Low reponders < 33% n = 18	Middle responders 33 – 66% n= 19	Good responders > 66% n = 19	p-value
Sociodemographic data				
Women (%)	72.2 (n=13)	78.9 (n=15)	73.7(n=14)	0.883
Age (yr)	47.2 (9.5)	43.0 (11.0)	41.8 (12.4)	0.309
Smoking status (%)	0 (n=0)	31.6 (n=6)	26.3 (n=5)	0.038 [a]
Food budget constraint (%)	16.7 (n=3)	21.1 (n=4)	5.3 (n=1)	0.364[a]
Follow up visit				
6-month	27.8 (n=5)	42.1 (n=8)	36.8 (n=7)	0.877
12-month	33.3 (n=6)	31.6 (n=6)	26.3 (n=5)	
24-month	38.9 (n=7)	26.3 (n=5)	36.8 (n=7)	
Anthropometric data				
BMI Before surgery(kg.m-^{2})	42.5 (5.4)	43.4 (5.8)	44.8 (6.8)	0.826
Body weight (kg)	95.4 (12.3)	87.2 (14.9)	81.2 (22.4)	0.050
BMI (kg.m-^{2})	33.8 (3.7)	31.2 (4.0)	29.2 (4.6)	0.005
% TWL	20.0 (4.4)	27.7 (2.8)	34.83 (4.9)	<0.001
Appetite sensations				
Mean hunger (mm)	17.2 (24.9)	15.4 (22.6)	30.4 (28.1)	0.149
Mean fullness (mm)	70.5 (31.7)	69.7 (27.6)	64.6 (25.7)	0.791
Mean desire to eat (mm)	18.1 (22.4)	20.2 (22.5)	28.0 (29.0)	0.444
Time since last meal (min)	117 (215)	117 (180)	168 (251)	0.719

Abbreviations: BMI: Body Mass Index; % TWL: Percentage of Total Weight Loss. Data are presented as mean (SD). p values are for ANOVA results; [a] indicates that Kruskal-Wallis one-way analysis of variance was used.
